# Author Correction: Prioritizing phylogenetic diversity captures functional diversity unreliably

**DOI:** 10.1038/s41467-019-08612-4

**Published:** 2019-02-04

**Authors:** Florent Mazel, Matthew W. Pennell, Marc W. Cadotte, Sandra Diaz, Giulio Valentino Dalla Riva, Richard Grenyer, Fabien Leprieur, Arne O. Mooers, David Mouillot, Caroline M. Tucker, William D. Pearse

**Affiliations:** 1 Department of Biological Sciences, Simon Fraser University, Burnaby, BC V5A 1S6 Canada; 2 Department of Botany, University of British Columbia, Vancouver, BC V6T 1Z4 Canada; 3 Biodiversity Research Centre, University of British Columbia, Vancouver, BC V6T 1Z4 Canada; 4 Department of Zoology, University of British Columbia, Vancouver, BC V6T 1Z4 Canada; 5 Biological Sciences, University of Toronto-Scarborough, Scarborough, M1C 1A4 Canada; 6 Ecology and Evolutionary Biology, University of Toronto, Toronto, ON M5S 3B2 Canada; 7 Instituto Multidisciplinario de Biología Vegetal, CONICET and FECFyN - Universidad Nacional de Córdoba, Casilla de Correo 495, 5000 Córdoba, Argentina; 8 Department of Statistics, University of British Columbia, Vancouver, BC V6T 1Z4 Canada; 9 School of Geography and the Environment, University of Oxford, Oxford, OX1 3QY UK; 10 Marine Biodiversity, Exploitation, and Conservation (MARBEC), UMR 9190, Université de Montpellier, Montpellier, 34095 France; 11 Australian Research Council Centre of Excellence for Coral Reef Studies, James Cook University, Townsville, QLD 4811 Australia; 12 Department of Biology, University of North Carolina-Chapel Hill, Chapel Hill, NC 27599-3280 USA; 13 Ecology Center and Department of Biology, Utah State University, Logan, UT 84322 USA

Correction to: *Nature Communications;* 10.1038/s41467-018-05126-3; Published online 23 July 2018.

The original version of this Article contained a plotting error in Fig. 3g. The Serranidae and Siganidae families were misplaced in the plotted phylogeny. This error has now been corrected in the PDF and HTML versions of the Article. For comparison, the original, incorrect version of Fig. 3g is presented below as Fig. 1. The authors thank P. Cowman for identifying the plotting error.

**Figure 1:**
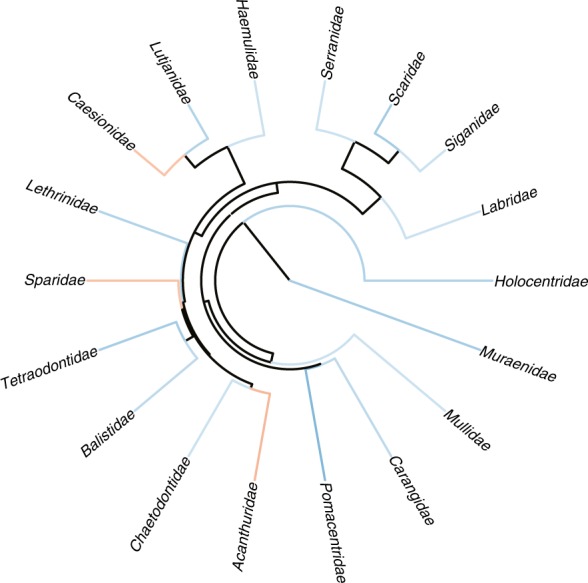
Fig. 1

