# Supplementary material for: Prioritizing phylogenetic diversity captures functional diversity unreliably
Source: Nat Commun. 2018 Jul 23;9:2888. doi: 10.1038/s41467-018-05126-3 (PMC6056549; doi:10.1038/s41467-018-05126-3)
Supplement: Supplementary file 1 — Supplementary Information [file 41467_2018_5126_MOESM1_ESM.pdf]

Supplementary Information for

Prioritizing phylogenetic diversity captures functional diversity unreliably

Mazel at al.

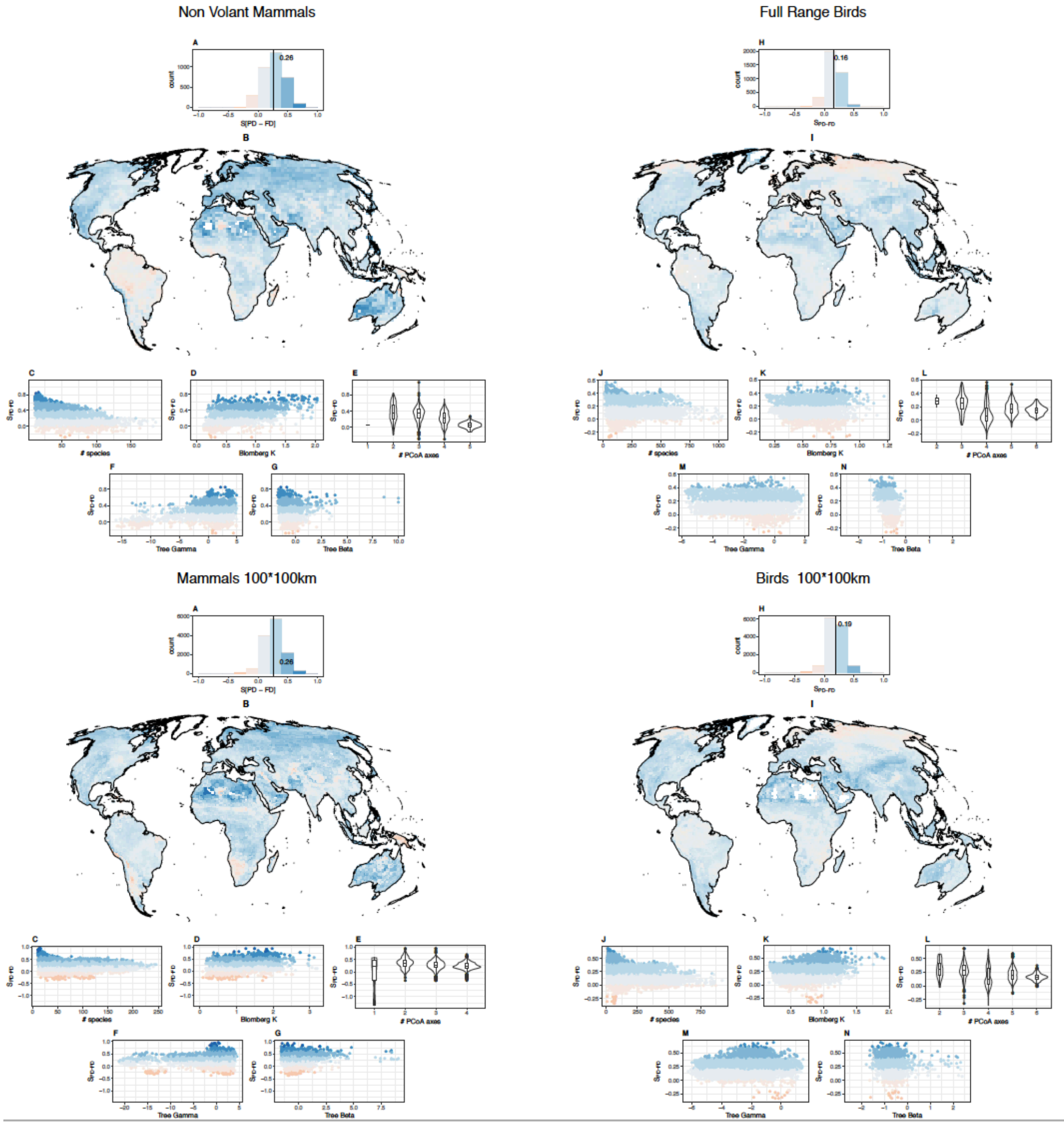

Supplementary Figure 1. Robustness of the assemblage results to variations grid cells definition and composition. This figure presents the same sets of panels for (i) non volant mammals only (i.e. excluding bats, A-G, top panels), (ii) using birds full ranges (and not breeding range only, H-N, top panels), and modifying the spatial scale of the grid cells (100*100km instead of 200*200 km) for (iii) mammals (A-G, bottom panels) and (iv) birds (H-N, bottom panels). For each of the four data types, the SD_{PD-FD} frequency distributions is presented in top panels along with its mean (vertical line) and the color code that is common to all panels, with blue indicating positive S_{PD-FD} (maximizing PD capture more FD than random). SD_{PD-FD} geographical distribution is presented in middle panels. Correlates of SD_{PD-FD} are presented in lower panels. In each grid cells, SD_{PD-FD} values are based on the mean over 1000 repetitions of random and PDmax set draw (there is only one maxFD set). The maps in this figure were generated making use of the R-package rasterVis and latticeExtra.

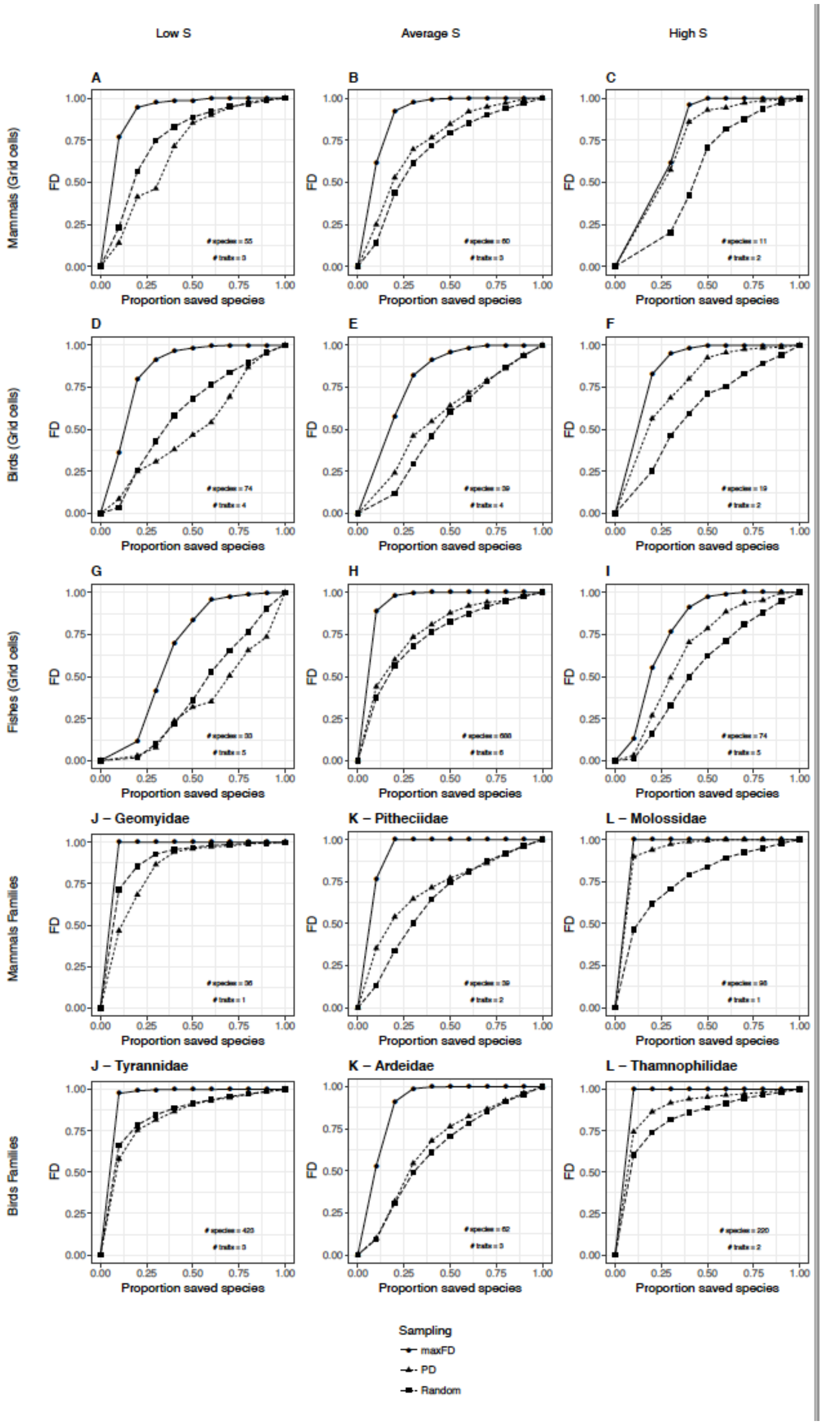

Supplementary Figure 2. Empirical sampling curves. The figure presents sampling curves (random, surrogate and optimal) as described in the figure 1 of the main text. Columns represent extreme (very low $SD_{PD\text{-}FD}$, left column; very high $SD_{PD\text{-}FD}$, right column) or average (mean $SD_{PD\text{-}FD}$, middle column) cases. Rows represent different species pools (Assemblages: rows 1-3; Clades: rows 4-5). Number of species and number of retained PCoA axes are depicted in each panel.

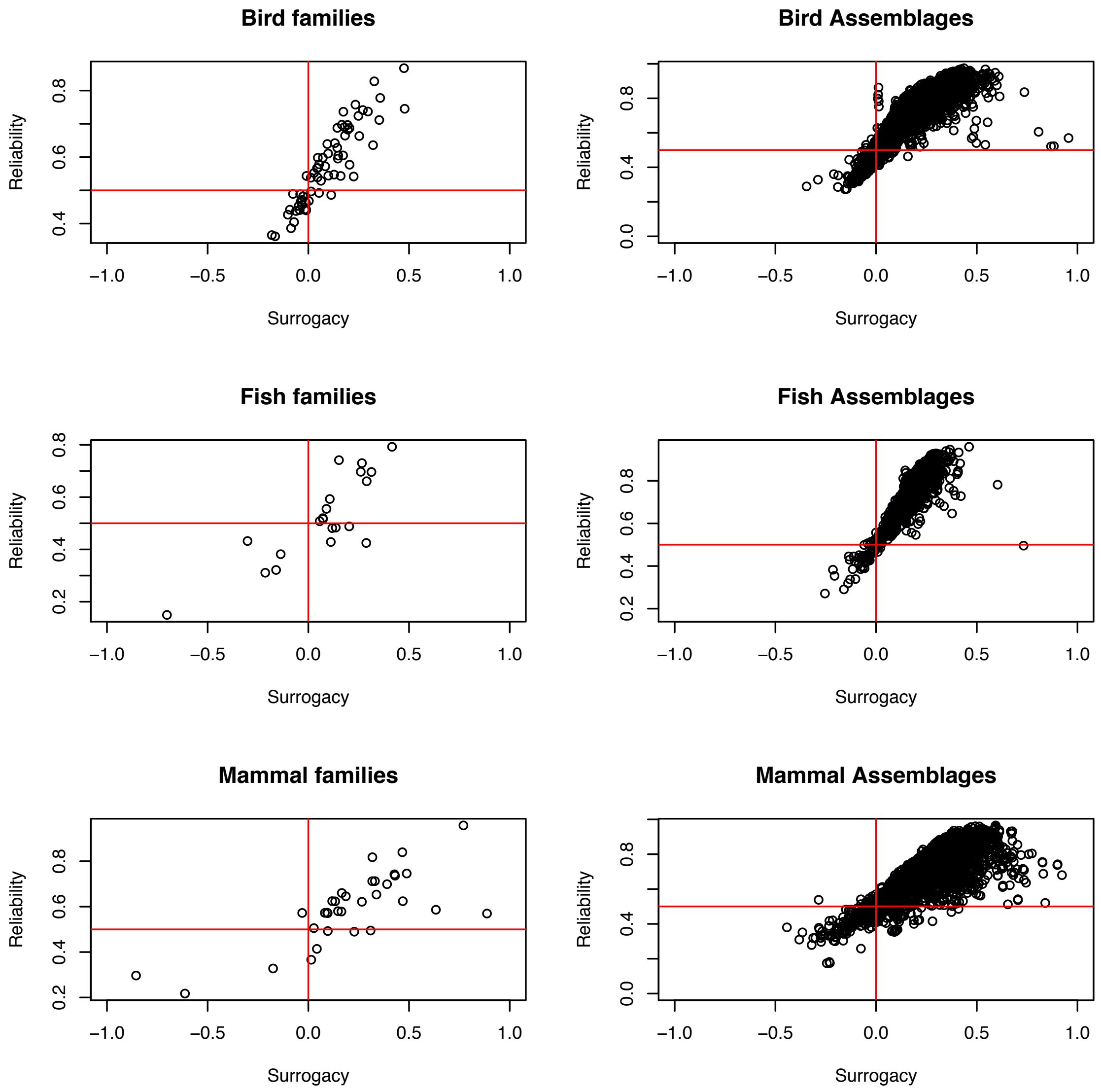

Supplementary Figure 3. The correlation between reliability of the maxPD strategy to yield more FD than a random strategy and surrogacy. For each species pool and for each % of selected species independently, the number of cases where $FD_{random}<FD_{maxPD}$ across the 1000 random *1000 maxPD sets combinations (i.e. 10^6 comparisons). We then averaged theses number across % of selected species to obtain the reliability metric. Plots depict the relationship between reliability and surrogacy (SD_{PD-FD}) for each data sets.

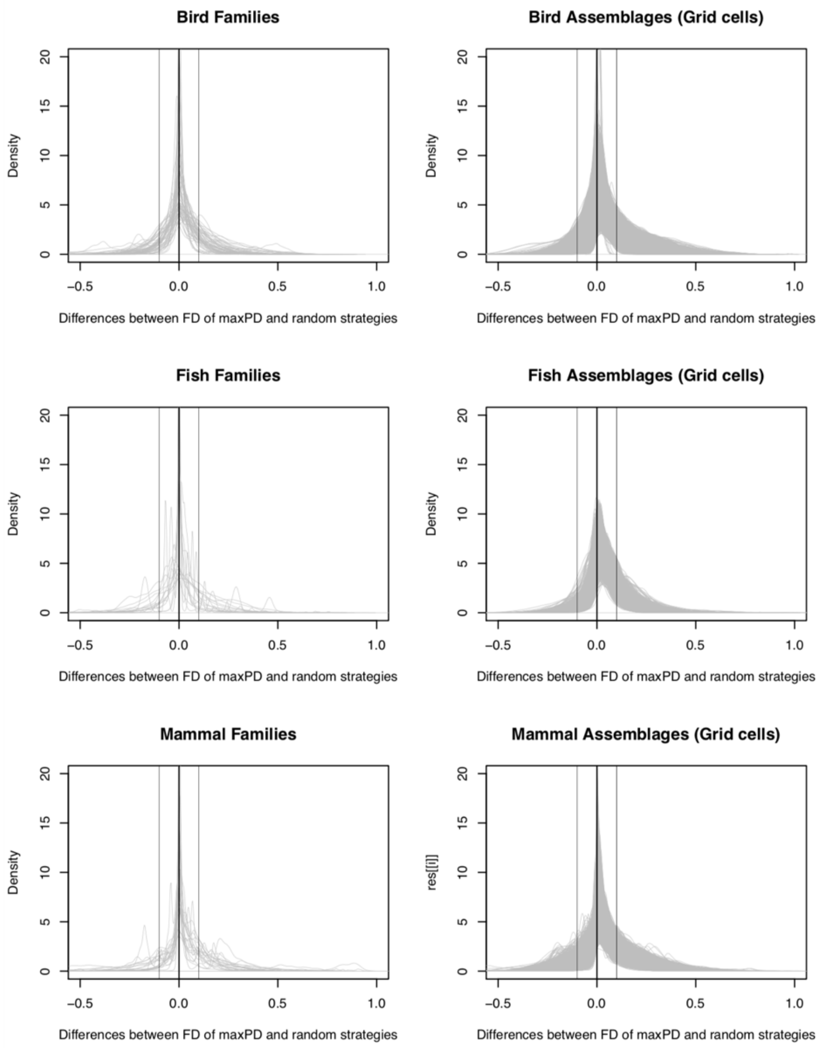

Supplementary Figure 4. Variability around the surrogacy estimate based on average FDs. This figure contrasts the variability of FD outcomes from the PD-maximization strategy to the random strategy. Instead of using only the mean FD outcomes of these two strategies (i.e. surrogate estimates), we present here all comparisons of individual FD computations for each species pool and for each % of selected species. In each of these individual cases, we subtracted the standardized FD (i.e. divided by maximal FD) obtained with a random strategy from the standardized FD obtained with a PD-maximization strategy (i.e. 1000 random *1000 maxPD sets combinations, 10^6 comparisons). Negative values represent cases where the random strategy yielded higher FD than the PD-maximization strategy whole positive values represent cases where the random strategy yielded lower FD than the PD-maximization strategy. The figure presents the fitted distribution of these values for each species pool independently, plotted together by dataset. Distributions tends to be shifted toward positive values because on average, the PD-maximization strategy is better than random at capturing FD. But this is not always the case: the distribution overlap with 0, maximizing PD is thus an unreliable strategy to capture FD. Vertical black bars represent values of -.1 ,0 and .1. This figure is complementary to supplementary Figure 3.

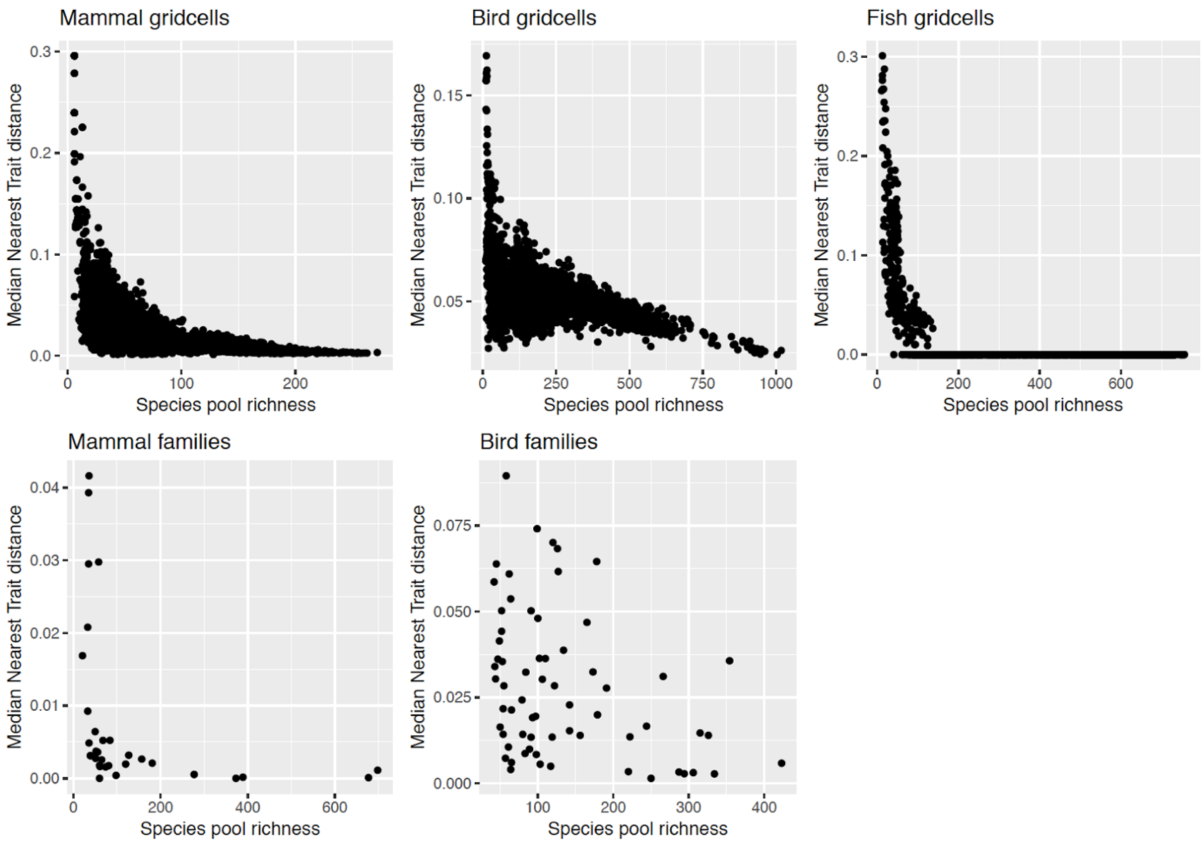

Supplementary Figure 5. Species rich assemblages and clades are more functionally redundant. The figure presents the correlation between species pool richness (X-axis) and median trait redundancy of the species in the pool (Y-axis, computed as the median of nearest trait distances between species).

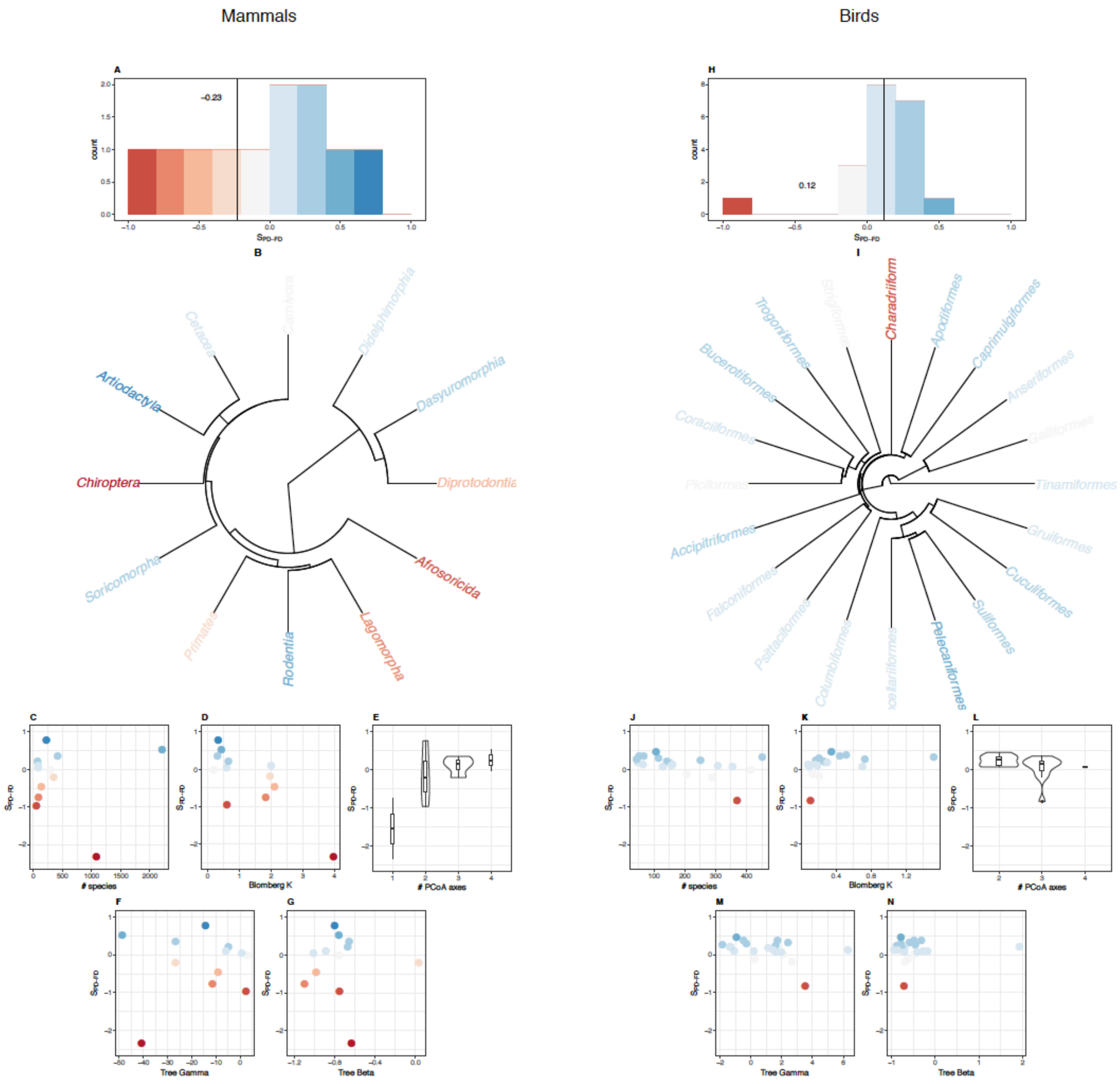

Supplementary Figure 6. Robustness of the clade-based results to variations of the taxonomic scale. The figure presents the distribution and correlates of $SD_{PD\text{-}FD}$ for mammals (panels A-G) and birds (panels H-N) across families. For each of the two groups, the $SD_{PD\text{-}FD}$ frequency distributions is presented in top panels (A and H) along with its mean (vertical line). The colour code that is common to all panels. $SD_{PD\text{-}FD}$ geographical distribution is presented in middle panels (B and I). Correlates of $SD_{PD\text{-}FD}$ are presented in lower panels (C-G and J-N). In each grid cells, $SD_{PD\text{-}FD}$ values are based on the mean over 1000 repetitions of random and maxPD set draw (there is only one maxFD set).

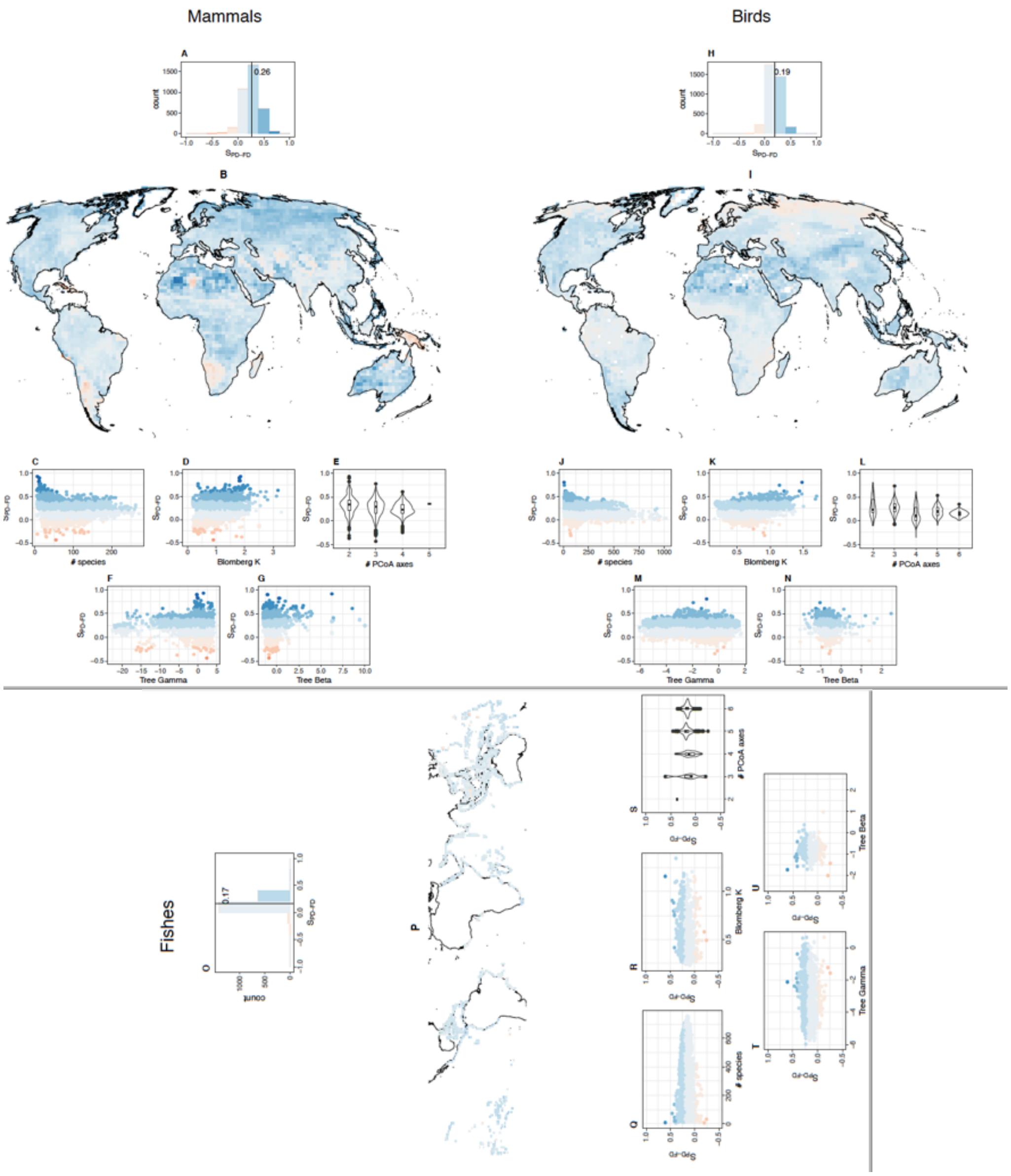

Supplementary Figure 7. PD as a surrogate of FD across space. The figure presents the distribution and correlates of $S_{PD\text{-}FD}$ for mammals (panels A-G), birds (panels H-N) and tropical fishes (O-U) separately across space. For each of the three groups, the $SD_{PD\text{-}FD}$ frequency distributions is presented in top panels (A, H and O, same as Fig. 2) along with its mean (vertical line) and the color code that is common to all panels, with blue indicating positive $S_{PD\text{-}FD}$ (maximizing PD capture more FD than random). $SD_{PD\text{-}FD}$ geographical distribution is presented in middle panels (B, I, P, same as Fig. 2). Correlates of $SD_{PD\text{-}FD}$ are presented in lower panels (C-G, J-N and Q-U). In each grid cells, $SD_{PD\text{-}FD}$ values are based on the mean over 1000 repetitions of random and PDmax set draw (there is only one maxFD set). The maps in this figure were generated making use of the R-package rasterVis and latticeExtra.

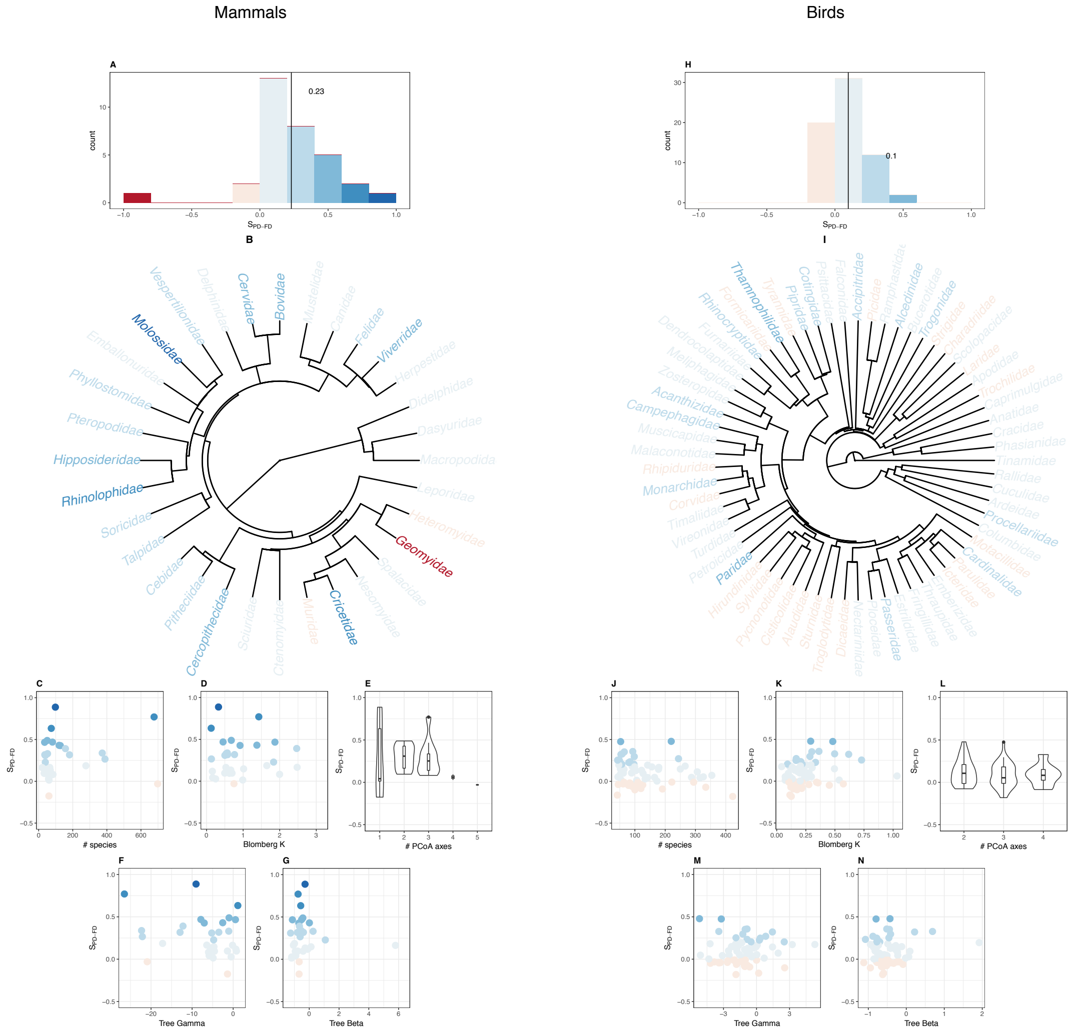

Supplementary Figure 8. PD as a surrogate of FD across clades. The figure presents the distribution and correlates of $SD_{PD\text{-}FD}$ for mammals (panels A-G) and birds (panels H-N) across families. For each of the two groups, the $SD_{PD\text{-}FD}$ frequency distributions is presented in top panels (A and H, same as Fig. 3) along with its mean (vertical line). The colour code that is common to all panels. $SD_{PD\text{-}FD}$ geographical distribution is presented in middle panels (B and I, same as Fig. 3). Correlates of $SD_{PD\text{-}FD}$ are presented in lower panels (C-G and J-N). In each grid cells, $SD_{PD\text{-}FD}$ values are based on the mean over 1000 repetitions of random and maxPD set draw (there is only one maxFD set).

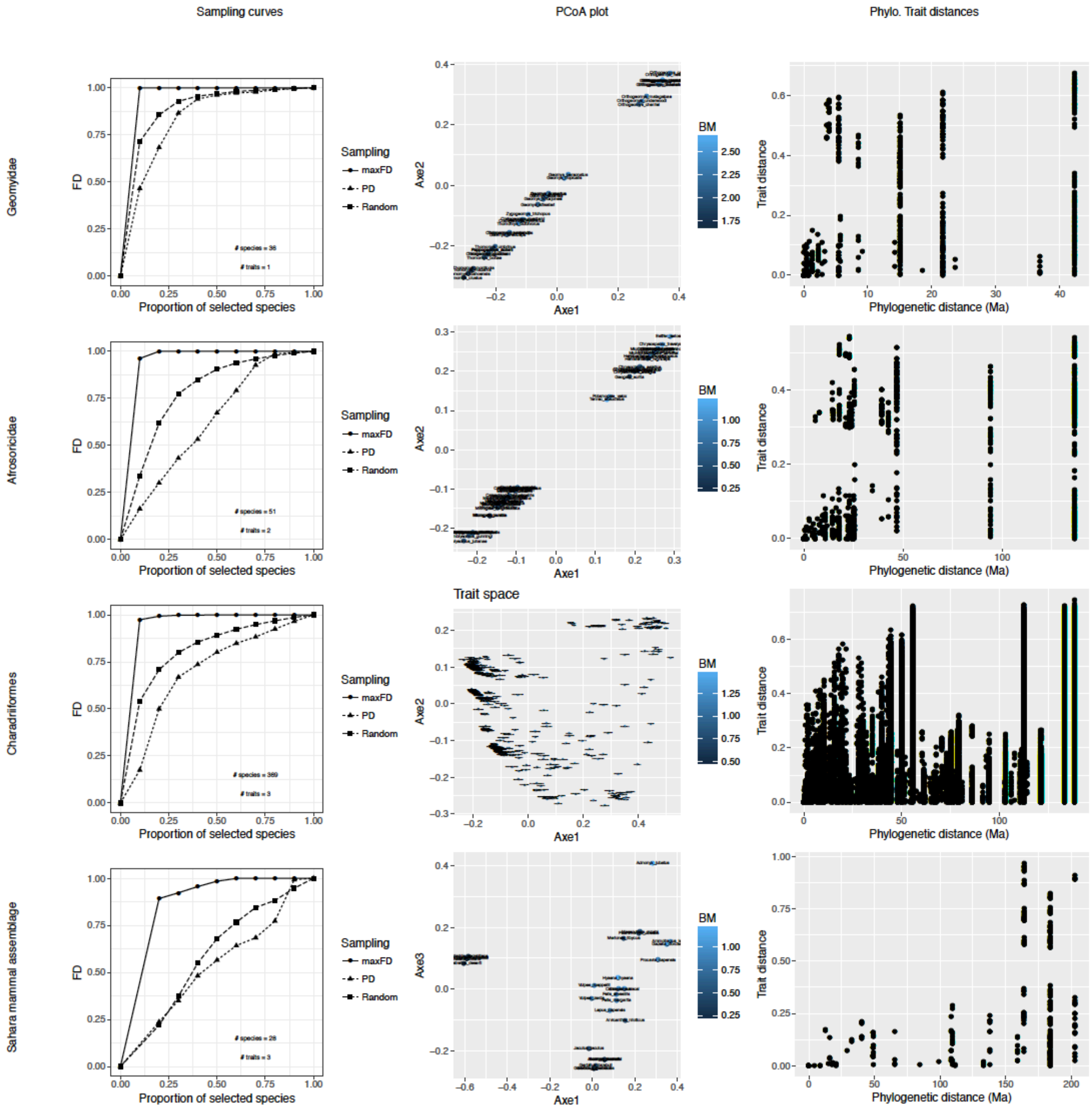

Supplementary Figure 9. Case examples when PD is doing worse than random at preserving FD. The figure depicts the sampling curves (column 1), the trait space as summarized by the PCoA axis (column 2, when only one axis has been retained, this axis is given in X and Y, BM indicates (log transformed and range standardized) body mass) and a plot contrasting phylogenetic and trait distances (column 3) for four different species pools (rows).

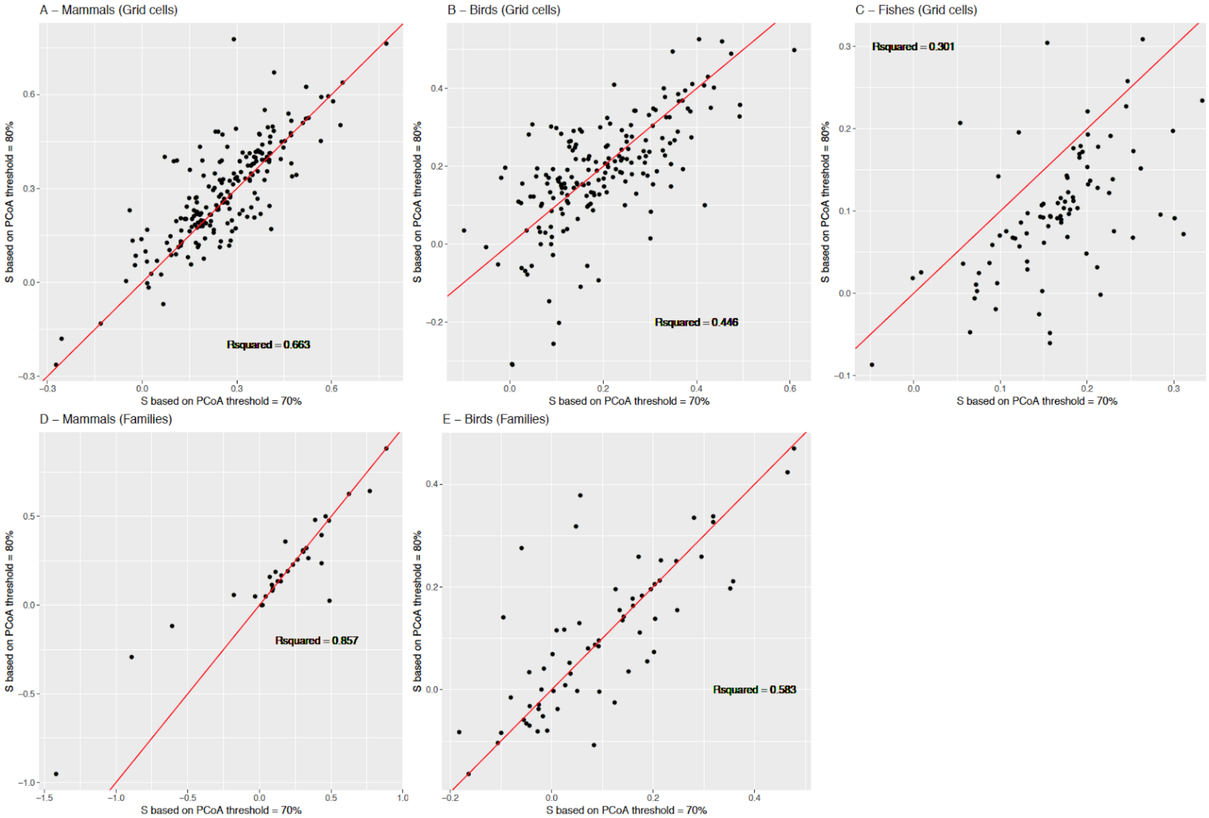

Supplementary Figure 10. Robustness of the results to variations of the number of retained PCoA axes. This figure depicts the correlation between SD_{PD-FD} computed with PCoA axes representing at least 70% of the initial variability (similar to the main text, X-axis) and SD_{PD-FD} computed with PCoA axes representing at least 80% of the initial variability (Y-axis). Because it was too intensive to calculate SD_{PD-FD} computed with PCoA axes representing at least 80% of the initial variability, only a representative subset of the total grid cells is given (see methods). The different panels correspond to different datasets (A: Mammal Grid cells; B: Bird Grid Cells; C: Fish Grid Cells; D: Mammal families; E: Bird Families).

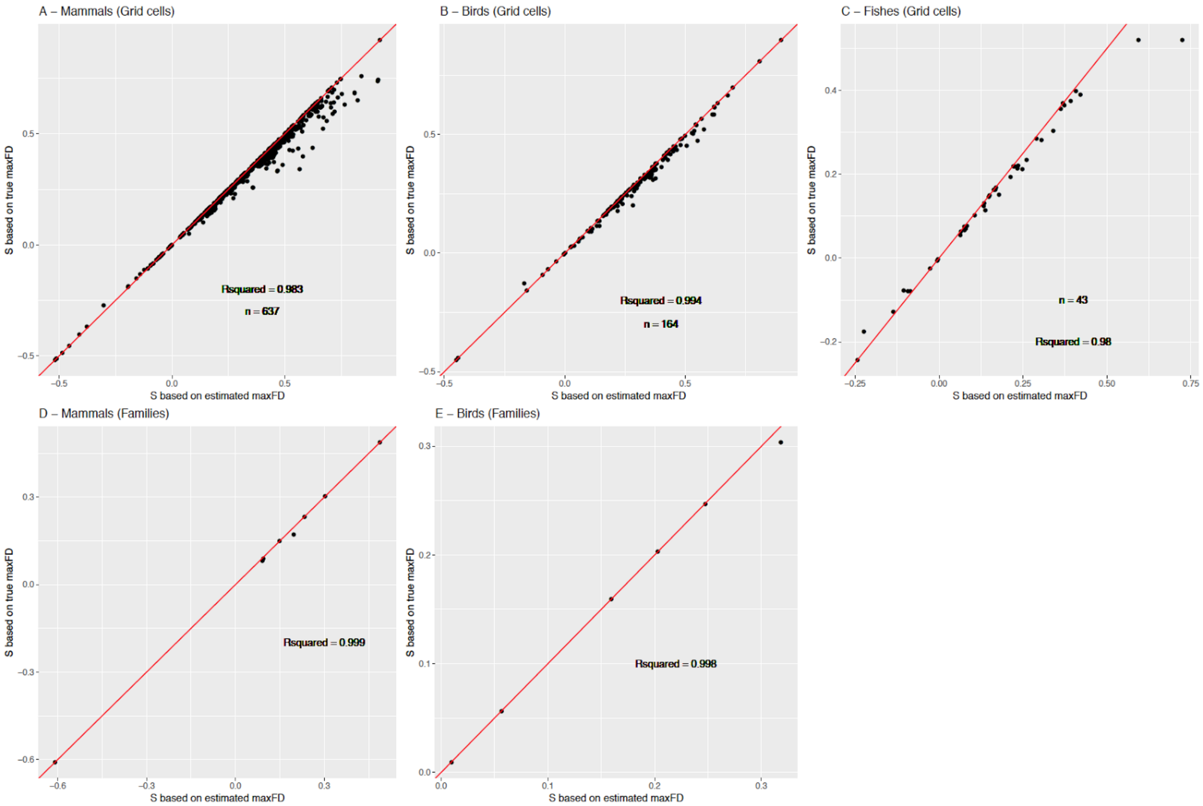

Supplementary Figure 11. Validation of the greedy algorithm used to delineate the maxFD set of species. The figure presents the correlation between $SD_{PD\text{-}FD}$ computed with the true optimal strategy and $SD_{PD\text{-}FD}$ computed with the greedy algorithm we developed. Because it was too computationally intensive to obtain the true optimal strategy for all gridcells, we only used a subset of gridcells. The different panels correspond to different datasets (A: Mammal Grid cells; B: Bird Grid Cells; C: Fish Grid Cells; D: Mammal families; E: Bird Families).

	Mean	Sd
Bird families	57.4	11.9
Bird Assemblages	70.5	13.6
Fish families	52	16.2
Fish Assemblages	74.2	9.8
Mammal families	58.5	16.4
Mammal Assemblages	70.3	13.1

Supplementary table 1. The reliability of the maxPD strategy to yield more FD than a random strategy. For each species pool and for each % of selected species independently, the number of cases where $FD_{random}<FD_{maxPD}$ across the 1000 random *1000 maxPD sets combinations (i.e. 10^6 comparisons). We then averaged theses number across % of selected species. Mean and standard deviations of theses metrics are given in the table.
