# Supplementary material for: Prioritizing phylogenetic diversity captures functional diversity unreliably
Source: Nat Commun. 2018 Jul 23;9:2888. doi: 10.1038/s41467-018-05126-3 (PMC6056549; doi:10.1038/s41467-018-05126-3)
Supplement: Supplementary file 2 — Peer Review File [file 41467_2018_5126_MOESM2_ESM.pdf]

Editorial Note: This manuscript has been previously reviewed at another journal that is not operating a transparent peer review scheme. The manuscript was considered suitable for publication without further review at *Nature Communications.*
